# Supplementary material for: Knowledge, attitudes, and practices towards Human Papilloma Virus and uptake of HPV vaccine: A protocol for a systematic review
Source: PLoS One. 2024 Nov 26;19(11):e0313887. doi: 10.1371/journal.pone.0313887 (PMC11594429; doi:10.1371/journal.pone.0313887)
Supplement: S2 Table — (DOCX) [file pone.0313887.s002.docx]

**S2 Table- Search strategy**

**Database:**
OVID Medline Epub Ahead of Print, In-Process & Other Non-Indexed Citations, Ovid MEDLINE(R) Daily and Ovid MEDLINE(R) 1946 to Present

| **#** | **Query** | **Results from 24 May 2024** |
| --- | --- | --- |
| 1 | exp Human Papillomavirus Viruses/ | 7,852 |
| 2 | HPV.mp. | 54,316 |
| 3 | Human Papillomavir*.mp. | 49,748 |
| 4 | 1 or 2 or 3 | 64,952 |
| 5 | exp Health Knowledge, Attitudes, Practice/ | 128,047 |
| 6 | Knowledge.mp. | 1,038,264 |
| 7 | Attitude.mp. | 331,345 |
| 8 | Practice.mp. | 1,285,237 |
| 9 | 5 or 6 or 7 or 8 | 2,320,349 |
| 10 | 4 and 9 | 7,946 |
| 11 | limit 10 to yr="2006 -Current" | 7,358 |
| 12 | exp Papillomavirus Vaccines/ | 10,557 |
| 13 | HPV vaccine.mp. | 6,411 |
| 14 | 12 or 13 | 12,154 |
| 15 | 4 or 14 | 65,611 |
| 16 | 9 and 11 and 15 | 7,358 |

exp Human Papillomavirus Viruses/
HPV.mp.
Human Papillomavir*.mp.
1 or 2 or 3
exp Health Knowledge, Attitudes, Practice/
Knowledge.mp.
Attitude.mp.
Practice.mp.
5 or 6 or 7 or 8
4 and 9
limit 10 to yr="2006 -Current"
exp Papillomavirus Vaccines/
HPV vaccine.mp.
12 or 13
4 or 14
9 and 11 and 15
